# Female Alms1-deficient mice develop echocardiographic features of adult but not infantile Alström syndrome cardiomyopathy

**DOI:** 10.1242/dmm.050561

**Published:** 2024-06-28

**Authors:** Eleanor J. McKay, Ineke Luijten, Sophie Broadway-Stringer, Adrian Thomson, Xiong Weng, Katya Gehmlich, Gillian A. Gray, Robert K. Semple

**Affiliations:** ^1^ Centre for Cardiovascular Science, University of Edinburgh, Edinburgh EH16 4TJ, UK; ^2^ Institute of Cardiovascular Sciences, University of Birmingham, Birmingham B15 2TT, UK; ^3^ Division of Cardiovascular Medicine, Radcliffe Department of Medicine and British Heart Foundation Centre of Research Excellence Oxford, University of Oxford, Oxford OX3 9DU, UK; ^4^ MRC Human Genetics Unit, Institute of Genetics and Cancer, University of Edinburgh, Edinburgh EH4 2XU, UK

**Keywords:** ALMS1, Alström syndrome, Alstrom syndrome, Heart, Cardiomyopathy, Ciliopathy, Primary cilia

## Abstract

Alström syndrome (AS), a multisystem disorder caused by biallelic ALMS1 mutations, features major early morbidity and mortality due to cardiac complications. The latter are biphasic, including infantile dilated cardiomyopathy and distinct adult-onset cardiomyopathy, and poorly understood. We assessed cardiac function of *Alms1* knockout (KO) mice by echocardiography. Cardiac function was unaltered in *Alms1* global KO mice of both sexes at postnatal day 15 (P15) and 8 weeks. At 23 weeks, female − but not male − KO mice showed increased left atrial area and decreased isovolumic relaxation time, consistent with early restrictive cardiomyopathy, as well as reduced ejection fraction. No histological or transcriptional changes were seen in myocardium of 23-week-old female *Alms1* global KO mice. Female mice with *Pdgfra-Cre*-driven Alms1 deletion in cardiac fibroblasts and in a small proportion of cardiomyocytes did not recapitulate the phenotype of global KO at 23 weeks. In conclusion, only female Alms1-deficient adult mice show echocardiographic evidence of cardiac dysfunction, consistent with the cardiomyopathy of AS. The explanation for sexual dimorphism remains unclear but might involve metabolic or endocrine differences between sexes.

## Teaser

**Summary:** *Alms1* knockout mice, although modelling many aspects of Alström syndrome, only weakly recapitulate the severe biphasic cardiomyopathy seen in humans. Adult female mice at 23 weeks show early systolic and diastolic dysfunction.

**Research Simplified**

Alström syndrome is a complex ultra-rare disorder in which cardiomyopathy – where heart muscle is weakened and often scarred – is a major cause of early death or transplantation. Mutations in the gene that codes for the ALMS1 protein have been implicated in heart failure in infants before any other signs of Alström syndrome have developed. Understanding how loss of the *ALMS1* gene causes cardiac abnormalities in Alström syndrome is a top priority for patients and researchers, and will help to develop potential therapies in the future. The authors used laboratory mice where the *ALMS1* gene was deleted in all cells of the body to investigate if they could mimic key features of Alström syndrome, including cardiac failure. Although no anatomical defects or cardiomyopathy were seen in these mice after birth, 23-week-old female mice lacking *ALMS1* showed evidence of heart stiffness and impaired pumping, but without all the severe signs of cardiomyopathy that are common in humans. Male mice of the same age lacking *ALMS1,* and mice of both sexes in which *ALMS1* was only deleted in specific cardiac cells, did not recapitulate any cardiac symptoms.

This study tracked laboratory mice through clinically relevant developmental stages to better understand the cardiac complications in humans with Alström syndrome. Further research into similar animal models can facilitate the development of therapies to treat this ultra-rare disease.

## INTRODUCTION

Alström syndrome (AS) is an autosomal recessive disorder caused by biallelic loss-of-function mutations in the *ALMS1* gene. The product of the *ALMS1* gene is a large, 460 kDa protein primarily localised to the centrosome and basal body of primary cilia. In keeping with this, cardinal features of AS include infantile rod-cone retinal dystrophy, sensorineural deafness, obesity and diabetes mellitus, which are common features of several so called primary ciliopathies. AS also features prominent cardiac complications, which are a major cause of early morbidity and mortality in the syndrome (Marshall et al., 2012).

Cardiac dysfunction occurs in ∼60% of patients with AS at some point. The natural history of cardiac manifestations of AS is complicated, however, with a biphasic pattern across the course of life. Dilated cardiomyopathy and congestive heart failure occurs in 43% of infants with AS, usually reported in the first 12 months of life. In some cases this is fatal but with treatment around three quarters of patients recover within 3 years (Marshall et al., 2005). The mechanism of infantile cardiac dysfunction in AS is poorly understood. One clue was offered by identification of biallelic *ALMS1* mutations in four infants who either died or underwent heart transplantation because of mitogenic cardiomyopathy, defined by persistent postnatal mitogenesis of cardiomyocytes (Louw et al., 2014; Shenje et al., 2014). Whether this was a sentinel presentation of AS is unknown but similar histological appearances have been reported in *Alms1*^GT/GT^ mice harbouring a gene trap in intron 13, resulting in global knockout (KO) of *Alms1* (Shenje et al., 2014). This remains the clearest potential explanation for the infantile heart failure of AS to date but corroboratory studies are needed. How loss of ALMS1 expression might induce persistent mitogenesis is unknown.

Approximately 30% of adults with AS develop heart failure that, for half of them, is fatal or requires a heart transplant (Marshall et al., 2005; Paisey et al., 2019). This appears independent of infantile cardiomyopathy (13% of patients develop both infantile and adult-onset cardiac dysfunction, and 18% of patients only develop adult-onset dysfunction) (Marshall et al., 2005; Corbetti et al., 2013; Baig et al., 2020b). Adult-onset cardiomyopathy in AS features myocardial hypertrophy and dilation with progressive fibrosis, and restrictive impairment of both ventricles (Marshall et al., 2012). Accelerated atherosclerosis and coronary artery disease (CAD) have also been reported (Jatti et al., 2012; Paisey et al., 2014; 2015; Baig et al., 2020b). Duration of diabetes in AS is predictive of aortic pulse wave velocity and, thus, cardiovascular events (Paisey et al., 2015), but no association between CAD and cardiac fibrosis has been found (Baig et al., 2020b). Of patients diagnosed with AS, 63% develop chronic kidney disease (CKD) (Baig et al., 2020a) and 30% have hypertension (Marshall et al., 2005; Waldman et al., 2018), both further possible drivers of CAD.

Cardiac pathology in AS may, thus, be a composite result of cardiomyocyte-autonomous developmental defect in infancy, with accelerated atherosclerosis and progressive fibrosis becoming prominent in young adulthood, driven in part by exogenous factors, such as diabetes, insulin resistance, dyslipidaemia, hypertension and impaired renal function. Unpicking the relative contributions of these processes to the strikingly poor cardiovascular outcomes in AS is difficult or impossible in human studies where they are generally admixed. This issue is highly important, as it will guide the choice of experimental treatments for future trials in AS. For example, the high prevalence of cardiac fibrosis suggests potential value for anti-fibrotic therapies (Baig et al., 2018); however, whether fibrosis is a cause of cardiac dysfunction or an epiphenomenon arising from a chronic repair process in AS is unknown.

Understanding the cardiac phenotype of *Alms1* KO mice may be clinically relevant to patients with AS and may also provide insights into common cardiac disease. In adults with AS, heart failure occurs alongside obesity and severe insulin resistance, and shows preserved ejection fraction (Edwards et al., 2015) – similar to heart failure with preserved ejection fraction (HFpEF) for which metabolic syndrome is a main risk factor.

Several *Alms1* KO mouse lines have been described. These lines recapitulate many key features of AS, including vision and hearing loss, obesity and insulin resistant diabetes (Collin et al., 2005; Arsov et al., 2006b; Li et al., 2007). Surprisingly, given its importance to patients with AS, no detailed evaluation of cardiac function in murine AS models has been offered to date. We, thus, set out to investigate cardiac phenotypes over the course of life using mice of a new *Alms1* global KO mouse model. To help distinguish heart autonomous and non-autonomous drivers of cardiac complications, we also studied mice with *Alms1* KO in only mesenchymal stem cells (MSCs), generated by utilising *Pdgfra-Cre* to drive KO (Roesch et al., 2008). This has been shown to drive recombination in cardiac fibroadipogenic precursor cells, such as cardiac fibroblasts − but only in 18% of cardiomyocytes (Lombardi et al., 2016). *Pdgfra*-*Cre* also drives recombination in preadipocytes (Jeffery et al., 2014; Krueger et al., 2014) and oligodendrocyte precursor cells in several brain regions (McKay et al., 2024) but not in liver or muscle (Jeffery et al., 2014; Krueger et al., 2014). By contrast, publicly available single nuclear RNA sequencing data show low expression of ALMS1/Alms1 within all cell types in the heart of healthy adult humans (Tucker et al., 2020) and mice (Speir et al., 2021).

## RESULTS

**Fig. 1.**
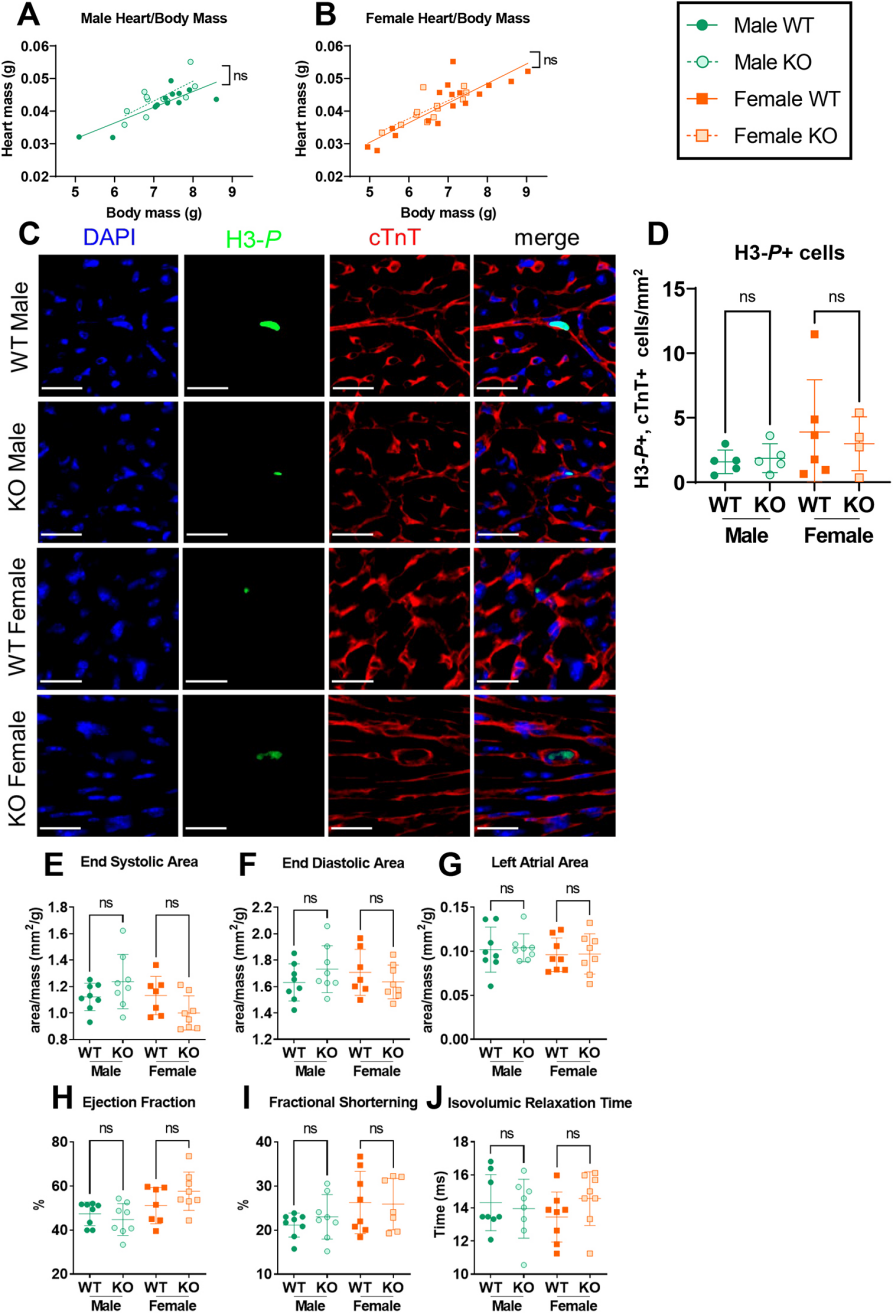
**Neither male nor female global *Alms1* knockout mice exhibit a cardiac phenotype at postnatal day 15.** (A,B) Linear regression of heart and body mass in wild-type (WT) and knockout (KO) male (A) and female (B) mice. Lines in linear regression graphs (A,B) represent lines of best fit. Comparisons between lines of best fit were undertaken by simple linear regression, with square brackets showing comparison of y intercepts. No significant change was seen between gradients. (C) Representative immunofluorescence images showing cardiac left ventricles of mice as indicated, stained for proliferative marker phosphorylated histone H3 (H3-*P*, green) and co-stained for cardiac muscle troponin T (cTnT, red). Nuclei were stained with DAPI (blue). Scale bars: 20 μm. (D) Quantification of images shown in C, showing percentage of cTnT-positive cells that also showed staining for H3-*P*. (E-J) Echocardiography data. Area values calculated from echocardiography (E-G) are normalised to total body mass, showing no changes in end-systolic or end-diastolic areas of the left ventricle, the left atrial area, and ejection fraction, fractional shortening or isovolumic relaxation times. Each data point represents an individual animal, with error bars in D-J representing the mean±s.d. Comparison between groups in panels D-J was undertaken using two-way ANOVA with Tukey's multiple comparisons test. Animals used per experiment were *n*=13, 11, 17 and 12 (A,B); *n*=5, 5, 6 and 4 (D); *n*=8, 8, 7 and 8 (E-J) for WT males, KO males, WT females and KO females, respectively. ns, not significant.

We first set out to assess whether our novel global *Alms1* KO mouse model can recapitulate the infantile cardiomyopathy of AS. One global *Alms1* KO mouse line harbouring a gene trap in *Alms1* intron 13 has previously been reported to show persistent mitogenesis in the heart and an increased heart to body mass ratio at postnatal day 15.5 (P15.5) (Shenje et al., 2014), a timepoint roughly equivalent to the developmental age at human birth. This is in keeping with the mitogenic cardiomyopathy described in four infants with biallelic *ALMS1* loss-of-function mutations (Shenje et al., 2014). We, thus, set out to seek corroboratory evidence for this in our newly generated global *Alms1* KO mouse line, while extending prior studies by characterising cardiac function by echocardiography. We elected to undertake functional studies at P15 both to replicate the study by Shenje et al. and because this corresponds to an age of high prevalence of infantile cardiomyopathy in AS. In contrast to the study by Shenje, however, we observed no changes in heart to body mass ratio in either male or female *Alms1* KO mice at P15 (Fig. 1A,B). Moreover, the number of cardiomyocytes staining positive for the cellular proliferation marker phosphorylated histone H3 (H3-*P*), which has previously been reported to be increased in the myocardium of *Alms1* KO mice at P15 (Shenje et al., 2014), did not differ between *Alms1* KO and wild-type (WT) littermates of either sex (Fig. 1C,D). Finally, no changes in several cardiac anatomical and functional measures obtained echocardiographically were seen. These included left ventricular mass, wall thickness and chamber dimensions (Fig. 1E-G, Fig. S1B,C), measures of systolic and diastolic function, longitudinal strain and ventricular dyssynchrony (Fig. 1H-J, Fig. S1D-H).

Consistent with the normal echocardiographic and histological appearances of hearts at P15, transcriptional analysis of myocardium for a panel of heart failure markers, i.e. actin alpha 1 (*Acta1*), myosin heavy chain beta (*Myh7*), and natriuretic peptides A and B (*Nppa* and *Nppb*, respectively) (Fig. S2A-D) normalised to *Gapdh* (e.g. Fig. S2E), showed no clear indication of cardiac dysfunction. An isolated increase of *Myh7* expression when comparing female WT and *Alms1* KO mice was inconsistent with expression of the other genes, and is of uncertain importance. Collectively, these findings provide no evidence of mitogenic or other forms of infantile cardiomyopathy in the new global *Alms1* KO mouse strain.

**Fig. 2.**
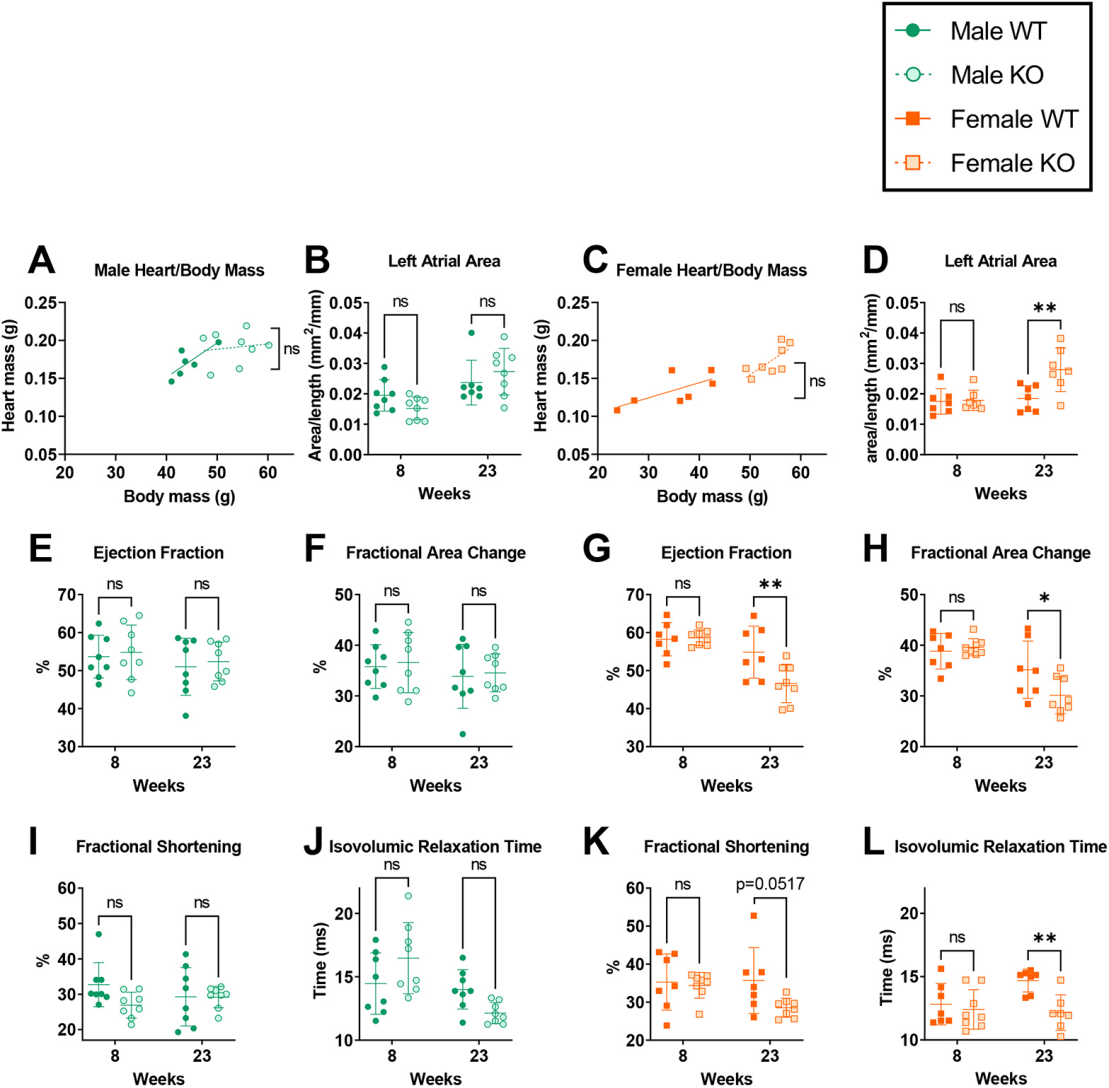
**Systolic and diastolic dysfunction develops with age in female but not male global *Alms1* knockout mice.** (A,C) Linear regression of heart to body mass in wild-type (WT) and knockout (KO) male (A) and female (C) mice at 24 weeks of age, showing that increased raw heart mass in *Alms1* KO mice is proportionate to total body mass. Lines in linear regression graphs (A,C) represent the of best fit. Comparisons between lines of best fit (A,C) were undertaken by simple linear regression, with square brackets showing comparison of y intercepts. No significant change was seen between gradients. (B, D-L) Echocardiography data of male and female WT and KO mice aged 8 and 23 weeks, showing no changes in male KO mice, but several structural and functional changes in female KO mice at 23 weeks of age. Each data point represents an individual animal. Left atrial area values (B,D) are normalised to nose–anus length. Error bars in B, D-L represent the mean±s.d., comparison between groups was undertaken using two-way ANOVA with Šídák's multiple comparisons test. Animals used per experiment were *n*=8, 8, 7 and 8 for WT males, KO males, WT females and KO females, respectively. Statistical significance: *P<0.05, **P<0.01, ****P<0.0001. ns, not significant.

We next sought to assess cardiac function in global *Alms1* KO mice during adulthood, i.e. at both 8 weeks and 23 weeks of age, with tissue collection at age 24 weeks. These age points correspond roughly to the second and fourth decade of human life, periods in which AS cardiomyopathy is commonly seen. The absolute heart mass of both males (Fig. 2A) and females (Fig. 2C) was increased at age 24 weeks; however, linear regression showed this increase to be proportionate to body mass, which is higher in AS and *Alms1* KO mice. No changes in echocardiographic anatomical or functional indices were observed in either male or female mice at 8 weeks of age (Fig. 2B,D-L, Fig. S4A-P). In female mice aged 23 weeks, however, the left atrial area – a well-established indirect indicator of diastolic cardiac dysfunction – was increased compared to that of WT littermate controls (Fig. 2D). Typically, diastolic dysfunction is also characterised by increased isovolumic relaxation time (IVRT) and/or decreased reverse longitudinal strain rate (Schnelle et al., 2018). However, in the more unusual case of restrictive diastolic dysfunction, decreased IVRT and/or increased reverse longitudinal strain rate have been shown to be good additional indicators in the diseased mouse heart when in conjunction with increased left atrial size (Schnelle et al., 2018). In agreement with clinical reports of restrictive diastolic dysfunction in patients with AS (Paisey et al., 2019), we found that IVRT was, indeed, decreased in female mice at 23 weeks of age (Fig. 2L), while reverse longitudinal strain rate showed no significant difference (Fig. S4O).

E/A ratio (the ratio between passive early diastolic flow rate across the mitral valve and late flow induced by atrial contraction) and E/e’ ratio (the ratio between E and the rate of early diastolic movement of the mitral valve) – of value in humans and larger rodents as further indices of diastolic function – could not be evaluated, because E and A waves are fused at physiological murine heart rates of 450-600 beats per minute (bpm), in line with prior findings in multiple other animal models (Schnelle et al., 2018). In male mice at 23 weeks of age, no significant difference in echocardiographic indices was seen, although a trend to increased left atrial area and reduced IVRT was observed (Fig. 2B,J).

Female global *Alms1* KO mice at 23 weeks compared to WT littermate controls, also showed several other cardiac functional changes suggestive of systolic dysfunction, including reduced ejection fractions and fractional ventricular area changes (Fig. 2G,H). Fractional shortening showed a trend towards reduction (Fig. 2K). Again, all these indices were unchanged in male mice (Fig. 2B,E,F,I,J). Moreover, left ventricle mass, wall thickness, cross-sectional areas, performance index, longitudinal strain and dyssynchrony were unchanged in both sexes at 23 weeks of age (Fig. S4A-P).

**Fig. 3.**
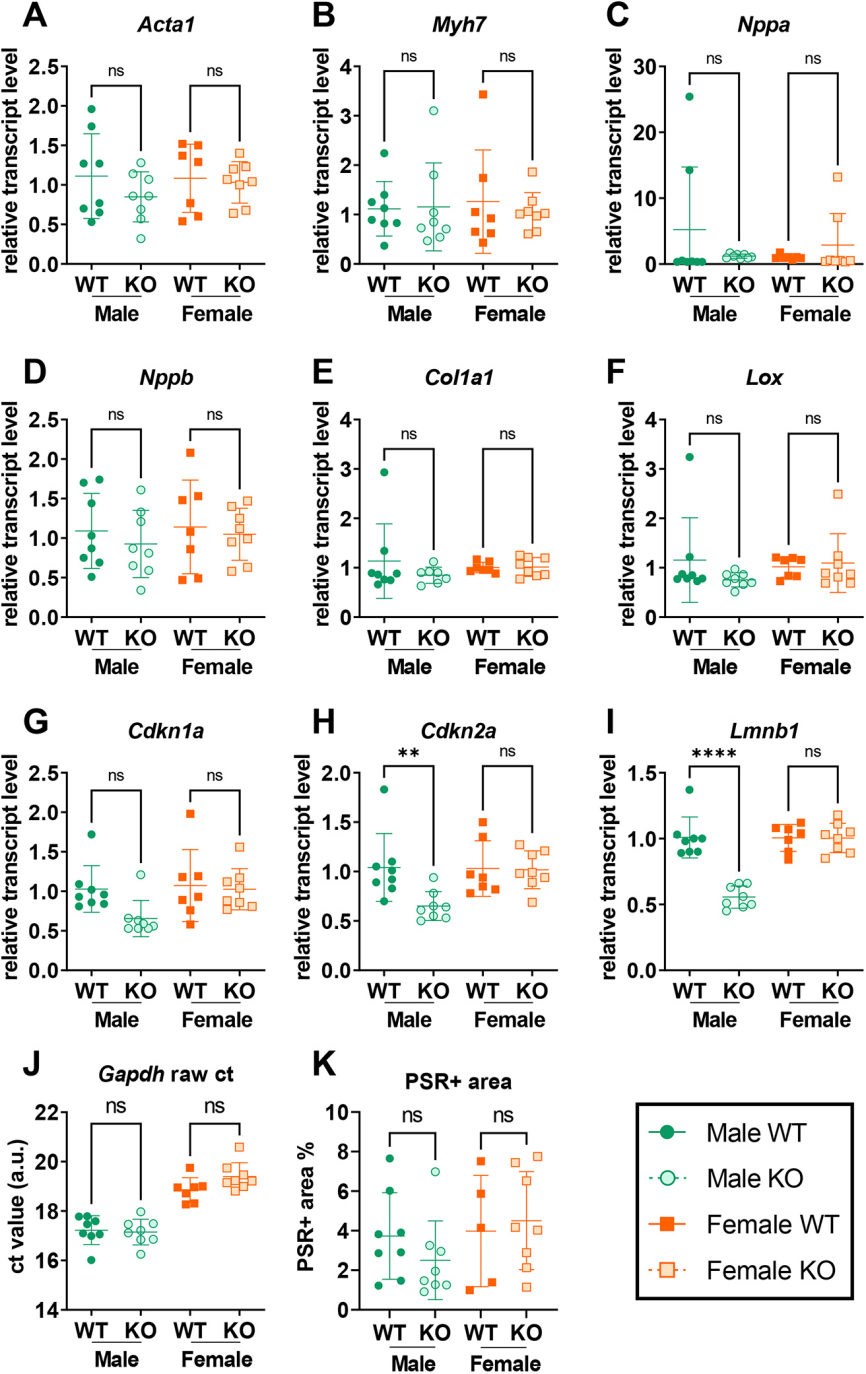
**Neither transcriptional nor histological correlates were found for the echocardiography phenotype of global *Alms1* knockout mice.** (A-I) Transcriptional analysis of hearts obtained at 24 weeks from wild-type (WT) and knockout (KO) male and female mice as indicated, showing minimal transcriptional changes in the cardiac tissue of Alms1 KO males and females. Data present crossing point (Cp) values of qPCR data (indicating the number of cycles which, in turn, indicate a significant increase in signal intensity) normalised to *Gapdh* values run in duplex. (J) An example of raw *Gapdh* Cp values from duplexed reactions. Data show that *Gapdh* transcript levels do not significantly differ between WT and KO animals of each sex. (K) Quantification of Picrosirius Red (PSR) staining by pixel thresholding, showing that *Gapdh* transcript levels do not significantly differ between WT and KO animals of each sex. Samples analysed from 24-week-old male and female *Alms1* knockout mice. Each data point represents an individual animal, with error bars representing the mean±sd. Comparisons between groups were performed using two-way ANOVA with Tukey's multiple comparisons test. Animals used per experiment were *n*=8, 8, 7 and 8 for WT males, KO males, WT females and KO females, respectively. Statistical significance: **P<0.01. ns, not significant.

Transcriptional analysis of the heart of male and female global *Alms1* KO mice at 24 weeks of age was undertaken next to evaluate markers of cardiomyopathy as before, with the addition of two genes associated with fibrosis, i.e. alpha-1 type I collagen (*Col1a1*) and lysyl oxidase (*Lox*), and three genes involved in cell cycle regulation and cellular senescence, i.e. cyclin dependent kinase inhibitor 1A (*Cdkn1a*), cyclin dependent kinase inhibitor 2A (*Cdkn2a*) and lamin B1 (*Lmnb1*). No change in expression of any of these genes was seen when comparing female global *Alms1* KO mice and WT littermates (Fig. 3A-I) when normalised to Gapdh (e.g. Fig. 3J) but, compared to male WT littermates global *Alms1* KO male mice did show decreased transcript levels of *Lmnb1* (Fig. 3I). However, although reduced *Lmnb1* expression is one marker of cellular senescence, *Cdkn1a* and *Cdkn2a* transcripts – which are typically elevated during senescence – were either decreased or showed a trend in this direction (*P*=0.15 for *Cdkn1a*), arguing against established senescence (Fig. 3G,H). There were no changes in the expression of genes typically associated with heart failure or fibrosis in male *Alms1* KO mice (Fig. 3A-F). Moreover, in keeping with unchanged transcript levels for fibrosis-associated genes, no increase in Picrosirius Red staining for fibrosis was seen (Fig. 3K).

**Fig. 4.**
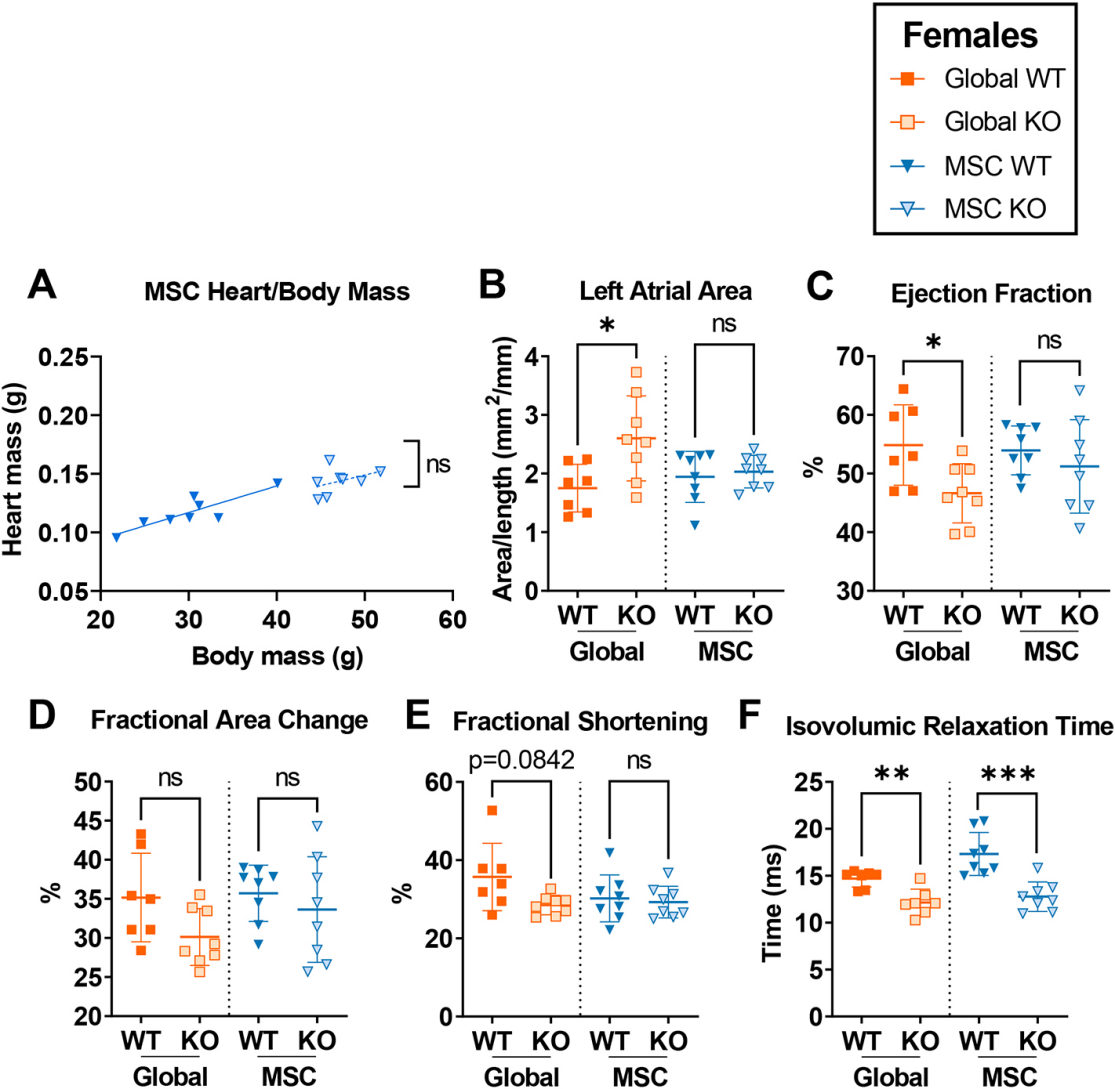
**Mesenchymal stem cell-specific *Alms1* knockout in female mice does not recapitulate the phenotype of global *Alms1* knockout.** All global knockout (KO) data were repeated according to those shown in Fig. 2 for comparison to mesenchymal stem cell (MSC)-specific *Alms1* KO. (A) Linear regression of heart to body mass in wild-type (WT) and knockout (KO) female mice at 24 weeks of age as indicated, showing that the increase in raw heart mass in *Alms1* MSC KO female mice is proportionate to total body mass. Lines in linear regression graphs represent lines of best fit. Comparisons between lines of best fit (A) was undertaken using simple linear regression, with square brackets showing comparison of y intercepts. No significant change was seen between gradients. (B-F) Data obtained from analysis of echocardiography in mice as indicated performed at 23 weeks of age, showing a failure of female *Alms1* MSC KO mice to recapitulate the cardiac phenotype seen in female *Alms1* global mice. Left atrial area (B) was normalised to nose–anus length. Each data point represents an individual animal, with error bars in B-F representing the mean±s.d. Global WT/KO and MSC WT and MSC KO experiments were performed with identical design at different times; this is reflected by the dotted line separating the two cohorts. Comparison between WT and KO in B-F was undertaken using unpaired two-tailed Student's *t*-test followed by Bonferroni correction for multiple testing. Animals used per experiment were *n*=7, 8, 8 and 8 for global WT, global KO, MSC WT and MSC KO, respectively. *P<0.05, **P<0.01 and ***P<0.001. ns, not significant.

Finally, to assess the contribution of different cell populations to the cardiac dysfunction seen in female mice at 23 weeks of age, the cardiac phenotype of mice in which *Alms1* KO was driven by the *Pdgfra* promoter, a marker of MSCs, was evaluated. As would be expected from loss of *Alms1* in cardiac fibroblasts and in ∼18% cardiomyocytes, *Alms1* myocardial transcript levels were significantly decreased but not lost completely in the heart of female MSC-specific *Alms1* KO mice (Fig. S5A). Similar to female global *Alms1* KO mice, heart mass was proportionate to body mass in female MSC-specific *Alms1* KO animals (Fig. 4A) but, in contrast to female global *Alms1* KO mice, female MSC-specific *Alms1* KO mice showed no differences in the left atrial area, ejection fraction, fractional area change or fractional shortening on echocardiography (Fig. 4B-E). The decrease in IVRT did remain in MSC-specific *Alms1* KO mice (Fig. 4F) but all other echocardiographic indices were unchanged (Fig. S5C-J), except the myocardial performance index, which was decreased (Fig. S5G).

## DISCUSSION

This study generated two *in vivo* models of AS, namely a new global KO and an MSC-specific *Alms1* KO, both derived from the same *Alms1* floxed parental line. Both were extensively characterised with respect to cardiac structure and function at multiple timepoints. Despite the prominence of cardiomyopathy in AS, and the high importance assigned to improving understanding of this by patients and families, such analysis has not been reported for any prior *Alms1* KO model. We found that, at 23 weeks of age, global *Alms1* KO female mice do show evidence of systolic dysfunction (reduced ejection fraction, fractional area change and fractional shortening), although neither myocardial performance index nor global longitudinal strain were significantly changed. An unusual form of diastolic dysfunction was suggested by the combination of increased left atrial area and decreased IVRT, which had previously been reported in models with restrictive diastolic dysfunction (Schnelle et al., 2018). This is in accord with clinical reports of cardiac dysfunction in AS (Paisey et al., 2019). However, the reverse longitudinal strain rate, another indicator of diastolic dysfunction, showed no significant difference.

Despite the echocardiographic abnormalities seen in female global *Alms1* KO mice at 23 weeks of age, no corresponding transcriptional or histological changes were detected in myocardial tissue, including no evidence of increased fibrosis or senescence. Nevertheless, our findings, collectively, do indicate mild combined systolic and diastolic dysfunction that is reminiscent of the restrictive cardiomyopathy of AS (Paisey et al., 2019), at least in adult female *Alms1* KO mice. As age is an important cofactor in the development of AS cardiomyopathy, it is plausible that more-pronounced cardiac dysfunction manifests itself at more-advanced age.

In contrast to findings in 23-week-old female KO mice, no phenotype was observed in male *Alms1* KO mice at the same age. This is notable, as males have been found to be more severely affected than females in several other mouse models of genetic cardiomyopathy (Du, 2004; Shinlapawittayatorn et al., 2022). No sexual dimorphism regarding cardiac pathology has been reported for human AS (Baig et al., 2020b). Diastolic dysfunction has been associated with non-cardiac drivers, such as hypertension, diabetes and obesity (Noll et al., 2020), and we have reported that the line of global *Alms1* KO mice used in this study is, like other global KO models described previously, obese and insulin resistant (McKay et al., 2024). The difference in these metabolic traits is much more pronounced between female WT and KO mice than between their male counterparts, and male KO mice are severely insulin resistant but not hyperglycaemic (McKay et al., 2024). This implicates metabolic differences as one possible cause of the sexual dimorphism we describe in the *Alms1* KO cardiac phenotype. In keeping with this, Heart Failure with preserved Ejection Fraction (HFpEF), which is associated with diabetes, is more prominent in females than males (Sotomi et al., 2021), while diabetes increases the risk of heart failure twice as much in females as in males (Kannel et al., 1974). Although no correlation has been found cross sectionally between metabolic dysregulation and cardiac dysfunction in human AS, numbers studied have been very small (Brofferio et al., 2017).

To narrow the search for the cellular origin of the cardiac phenotype of older female global *Alms1* KO mice, we also studied female mice with *Alms1* KO driven by *Pdgfra*-*Cre*. This has been shown to delete *Alms1* in a raft of MSCs, including cardiac fibroadipogenic precursors, while largely sparing cardiomyocytes (Lombardi et al., 2016). In this current study, the indices of systolic and diastolic dysfunction seen in female global *Alms1* KO mice had not been recapitulated by female MSC-specific *Alms1* KO mice. Significantly, these conditional KO mice do also have insulin resistance and diabetes (McKay et al., 2024). This is in contrast to the suggestion that the sexually dimorphic cardiomyopathy in female *Alms1* KO mice is purely metabolically mediated and, instead, indicates that cardiomyocyte-autonomous *Alms1* deficiency plays an important and, perhaps, dominant role. Cardiomyocyte-specific *Alms1* KO mice could, therefore, be used to confirm this in future.

One limitation of our study is that confirmation of complete loss of Alms1 protein was impossible, as no specific antibody against murine Alms1 is currently available. We did, however, confirm a frameshift deletion of exon 7 at both genomic DNA and cDNA level. Lack of protein-level KO confirmation is true also for prior KO models and, moreover, non-cardiac features of AS are well modelled and consistent across all AS mouse models. On this basis, we think the possibility that our findings are confounded by residual *Alms1* expression is negligible. A further limitation of our study is that blood pressure was not measured. *Alms1* KO rats are reported to be hypertensive, attributed to altered tubular trafficking of the Na+/K+/2Cl− (NKCC) co-transporter, and hypertension is observed in ∼30% of patients with AS (Marshall et al., 2005; Edwards et al., 2015; Brofferio et al., 2017). It is, thus, highly plausible that our new KO line, too, features hypertension and, therefore, future assessment of hypertension in this model is warranted.

We did not find evidence of cardiomyopathy in *Alms1* KO mice at P15, which is at odds both with the previous demonstration of increased heart/body mass ratio and persistent cardiomyocyte mitosis in *Alms1^GT/GT^* mice, and with mitogenic cardiomyopathy in four infants carrying biallelic *ALMS1* mutations (Shenje et al., 2014). Given the good concordance of several important phenotypic features of AS among different *Alms1* KO mice models, it was surprising that this early cardiac phenotype could not be replicated. The discrepancy between studies of AS-related infantile cardiomyopathy might relate to the genetic background, as Shenje and colleagues used mice with a mixed 129/C57BL6/J background. Genetic background has been shown to influence other murine cardiac phenotypes (Shah et al., 2010; Riehle and Bauersachs, 2019; Hart et al., 2022), while the incomplete penetrance (43%) of infantile cardiomyopathy in AS also argues that environmental or background genetic factors may play a role in development of AS cardiomyopathy in humans. Cardiomyocyte binucleation is typically complete by P10 in WT mice (Shenje et al., 2014), and it remains possible that a difference in the trajectory of cardiomyocyte maturation would have been discerned, if an earlier timepoint had been studied. Indeed, infantile cardiomyopathy in patients with AS commonly spontaneously recovers, in keeping with a self-limiting alteration of maturation kinetics. Future studies of *Alms1* KO mice incorporating multiple early postnatal timepoints may, thus, be informative.

*In toto*, although we detected early cardiac abnormalities in 23-week-old female global *Alms1* KO mice, this study demonstrates that mice do not faithfully replicate the severe biphasic cardiomyopathy common in human AS. This might reflect a fundamental inter-species difference in the consequences of *Alms1* loss, therefore, undermining the future use of mouse models for its study. Alternatively, it might reveal the relative contributions of different pathogenic mechanisms to heart failure in human AS. Two pertinent differences between C57BL6/N AS model mice and humans diagnosed with AS are that the mice are neither extremely prone to fibrosis (Walkin et al., 2013) nor to atherosclerosis. The absence of a severe cardiac phenotype in mice could, thus, be interpreted as strengthening the case that accelerated atherosclerosis and/or pathological abnormalities in remodelling in the face of ischaemia, and/or excess fibrosis are the dominant drivers of failure in adult patients with AS.

Several other murine models of cardiomyopathy model human disease poorly (see e.g. Vignier et al., 2009; Ehsan et al., 2018; Jiang et al., 2021). Several measures are worthy of consideration in future to increase the value of the *Alms1* KO mouse as a cardiac disease model. These include increasing the age of the mice studied, as age is an important factor in development of diastolic dysfunction (Riehle and Bauersachs, 2019; Noll et al., 2020), or using pharmacological stressors, such as angiotensin II or adrenergic agonists (Pilz et al., 2022) to ‘unmask’ cardiac dysfunction. More-specific options to gain insights into AS heart failure pathogenesis include examining *Alms1* KO on either an atherosclerosis-prone (e.g. *Apoe^−/−^* or *Pcsk9^−/−^*) or fibrosis-prone (S129S6) genetic background. Our findings, however, underline the value of alternative experimental models of the cardiac complications of AS including, for example, induced pluripotent stem cell-derived cardiomyocytes. Continuing to optimise models of cardiac complications of AS will be crucial not only for unpicking and more-effectively targeting devastating complications of an ultra-rare disease but may also yield prismatic insights into the role of complex multi-morbidities (e.g. diabetes, obesity, kidney and liver impairment) in more-common conditions, such as HFpEF.

## MATERIALS AND METHODS

### Mouse strains and experimental protocols

*Alms1*^tm1c(EUCOMM)Hmgu^ mice with *loxP* sites flanking exon 7 were generated by and purchased from genOway (Lyon, France). Excision of the floxed *Alms1* exon 7 yielded a premature stop codon in exon 8. CAG-Cre mice (Sakai and Miyazaki, 1997) were a gift from Dr Matthew Brook (Centre for Cardiovascular Science, University of Edinburgh, Edinburgh, UK) and used to generate a global *Alms1* KO. *Pdgfra*-Cre mice (Roesch et al., 2008) were purchased from The Jackson Laboratory (Strain #013148) and used to KO *Alms1* in mesenchymal stem cells (MSCs). Although no reliable antibody against murine Alms1 was available to prove the absence of Alms1 protein in the KO mice generated, recombination and loss of exon 7 of the *Alms1* transcript was confirmed both in genomic DNA (by Transnetyx, Cordova, TN, USA), and in cDNA from heart (Fig. S1A), liver and adipose tissue (McKay et al., 2024) by qPCR, as detailed below under ‘Gene expression analysis’. This is in line with previously published murine Alms1 KO models (see Table 1).

**Table 1.**
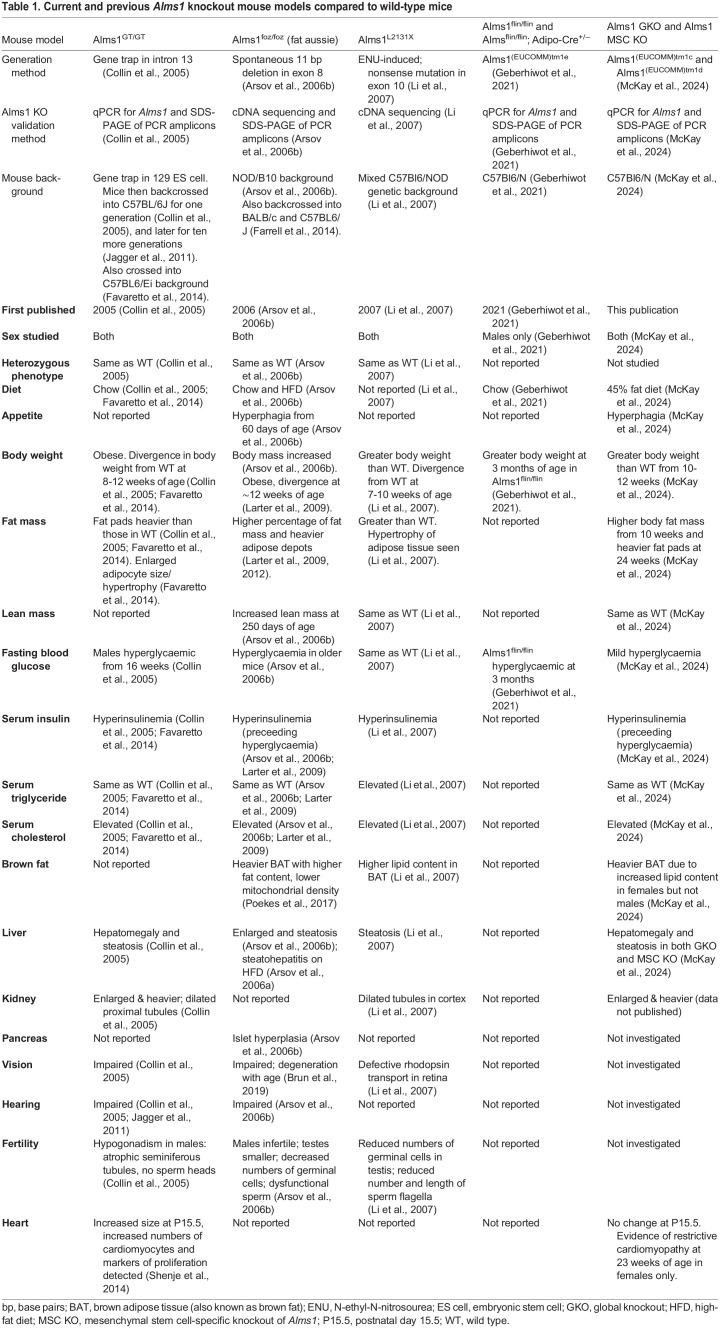
Current and previous *Alms1* knockout mouse models compared to wild-type mice

All mice were purchased and maintained on a C57BL/6N background, confirmed by single-nucleotide polymorphism profiling (Transnetyx). Genotyping was performed by Transnetyx using real-time PCR in all cases, except neonatal studies in which genotyping was performed in-house. In-house genotyping was performed by DNA gel electrophoresis following PCR amplification. One common forward primer (5′-ATACCACCACACCTGGGAGG-3′) and two reverse primers were used: reverse 1 (sequence contained in loxP flanked region; 5′-CACCATGTAAACACTAGAAATAGAACCCAGGTC-3′) and reverse 2 (5′-GCCAGGAGGAGCAAGACAAT-3′). Presence of WT *Alms1* results in generation of a 366 bp fragment by forward and reverse 1 primers. Excision of the loxP flanked sites results in the generation of a 306 bp fragment by forward and reverse 2 primers.

Mice were group-housed in individually ventilated cages at the biological research facility at the University of Edinburgh. A 12-h light/dark cycle (lights on at 0700 and off at 1900) and controlled temperature/humidity (19-21°C/50%) were maintained. Until 6 weeks old, mice had *ad libitum* access to standard chow (CRM, Special Diet Service), then replaced by 45% fat diet (D12451, Research Diets) to exacerbate systemic IR and any propensity to HFpEF, and to allow correlation with prior detailed metabolic studies undertaken on the same diet (McKay et al., 2024). Mice were single housed from 13 weeks of age. All experimental protocols were approved by the University of Edinburgh Biological Science Services and performed in compliance with the UK Home Office Scientific Procedure (Animals) Act 1983. Echocardiography was performed by a single experienced operator at Edinburgh Preclinical Imaging.

### Echocardiographic studies

For echocardiography mice were anaesthetised with 4% isoflurane in 100% oxygen for induction, with maintenance using 1-2% isoflurane. Animals were placed supine on a heated imaging table with paws attached to electrocardiogram (ECG) electrodes. Heart rate never significantly differed between genotypes and was maintained at 450-550 beats per minute (bpm), (Figs S1I, S4Q,R, S5K). Body temperature measured rectally was maintained at 37±0.5°C by using a heated table and heating lamp. Chest hair was removed using depilatory cream (Nair hair removal cream, Church & Dwight, London, UK) and ultrasonography was performed using a VisualSonics Vevo 3100 high frequency ultrasound imaging system (FUJIFILM VisualSonics). An MX550D transducer was used except for obese mice at 23 weeks, where a lower frequency MX250 transducer with increased scan depth was required for Doppler measurements. To study mice at P15.5, copper tape was used to extend the electrode pad to enable heart rate measurement, and an extra small rectal temperature probe was employed.

For left-ventricle-function assessment, electrocardiogram-gated Kilohertz visualisation (EKV) (Moran et al., 2013) was applied on the parasternal long-axis (PSLA) view and for M-mode on the parasternal short axis (PSAX) view at left ventricle midpoint with papillary muscles at 2 and 4 o'clock. Left atrium measurements were performed on EKV-modified right PSLA views. Doppler measurements of left ventricle inflow and outflow were obtained in the apical four-chamber view.

### Electrocardiographic analysis

Analysis of echocardiographic data was performed using Visualsonics Vevo LAB 5.71 software (FUJIFILM VisualSonics). End-systolic and end-diastolic area, ejection fraction, fractional area change, left ventricle mass and average wall thickness were calculated using the PSLA EKV data. End-systolic and end-diastolic area and ejection fraction were calculated using the automated artificial-intelligence software AutoLV (Grune et al., 2019). Fractional area change, left ventricle mass and average wall thickness were calculated after manual tracing of the contours of PSLA epicardial and endocardial area and axis length at systole and diastole, as indicated by AutoLV. Fractional shortening was calculated from M-mode images using AutoLV. Modified right-side PSLA EKV was used to manually measure left atrium area, choosing the time frame immediately after the mitral valve closure. Isovolumic relaxation time (IVRT), isovolumic contraction time (IVCT) and ejection time (ET) were all calculated by manual analysis of Doppler ultrasound data; three consecutive annotations for each index were made. IVRT measures the time between aortic valve closing and the mitral valve opening. Myocardial performance index (MPI) was calculated as the sum of IVRT and IVCT divided by ejection time. Finally, the ‘Vevo Strain’ function of the Vevo LAB software was used to analyse PSLA EKV, generating values for global longitudinal strain (GLS), reverse longitudinal strain rate (rLSR) and ventricular dyssynchrony. Ventricular dyssychrony was calculated as the standard deviation of strain across the six panels generated by ‘Vevo Strain’.

For normalisation of size-dependent measurements (left ventricle mass, ventricle and atria areas), the nose–anus length and tibia length were measured using a digital calliper. Nose–anus length was measured when animals were anaesthetised for echocardiography at 23 weeks of age. Tibia length was measured following dissection at 24 weeks of age. As tibia length was shorter in male and female global *Alms1* KO animals (Fig. S3A), nose–anus length – which did not differ by genotype (Fig. S3B) – was used for normalisation. For normalisation of neonatal echocardiography, body mass was used, which did not differ between experimental groups.

### Tissue studies

Mice were culled by cervical dislocation under isoflurane anaesthesia. Dissected hearts were rinsed in phosphate-buffered saline (PBS), and weighed after blotting of excess fluid. Whole neonatal hearts were fixed in 4% paraformaldehyde (PFA) or snap-frozen in liquid nitrogen. The bottom 1/5^th^ part (apex) of the heart of adult mice was fixed in 4% PFA, and the middle 2/5^th^ part (ventricles) snap-frozen in liquid nitrogen. Tibia of adult mice were also collected.

For histological analysis, fixed cardiac tissue was paraffin-embedded and 5 μm sections cut. For Picrosirius Red (PSR) staining, slides were dewaxed in xylene, rehydrated in decreasing concentrations of ethanol [20 s each in absolute (x3), 95% (1x), 80% (1x), 70% (1x) ethanol] and washed in tap water before incubation in direct Red Picric acid solution for 2 h, washing, dehydrating in ethanol [20 seconds each in 70% (1x), 80% (1x), 95% (1x), absolute (3x)], clearing in xylene, and mounting. Immunofluorescence staining of neonatal heart sections was performed using the Leica BOND-III staining system (Leica Biosystems) at room temperature, with reagents detailed in Table S1. Sequential staining for phosphorylated histone H3 (H3-*P*), cardiac muscle troponin T (cTnT) and wheat germ agglutinin (WGA) was performed, with antibodies and conditions used detailed in Table S2. Washing with TBS-T buffer (1x Tris-buffered saline with 0.1% Tween 20) was performed between steps. For H3-*P*, antigen retrieval was by incubation in Bond Epitope Retrieval ER1 Solution (Leica Microsystems) for 20 min before peroxide blocking, followed by blocking in diluted goat serum diluted 1:5 for 10 min. Antibody against phosphorylated histone H3 (H3-*P*) was incubated for 60 min before incubation with goat anti-rabbit IgG HRP-conjugated secondary antibody for 30 min. Finally, tyramide signal amplification was performed with addition of tyramine substrate FITC green opal 520 see Table S1 for further details). Next, cTnT staining was performed. First, antigen retrieval was performed by incubation in Bond Epitope Retrieval ER1 Solution for 10 min. Peroxide blocking was performed for 10 min, followed by serum blocking for 10 min using blocking solution from the Mouse on Mouse Polymer IHC Kit (Abcam; see Table S1 for further details). Incubation with anti-cTnT antibody was for 60 min before addition of the secondary antibody-bound polymer from the Mouse on Mouse Polymer IHC Kit (Abcam) for 30 min. Finally, the tyramide substrate Blue CY5 Opal 650 was added. Next, WGA staining was performed. Antigen retrieval was performed by incubation in Bond Epitope Retrieval ER1 Solution for 10 min. Peroxide blocking was performed for 10 min before serum blocking in goat serum diluted 1:5 for 10 min. Rhodamine-conjugated WGA diluted 1:75 was then incubated for 60 min. Finally, nuclear counterstaining was performed by incubation with DAPI, diluted 1:1000. All histological slides were imaged by using a Zeiss Axioscan.Z1 with Zen2.6 software.

### Gene expression analysis

RNA was extracted from snap-frozen heart tissue using the Qiagen RNeasy Fibrous Tissue Mini Kit after homogenisation in 2 ml tubes containing 2.8 mm ceramic beads stored on dry ice, using the Omni Bead Raptor 24 Elite with the pre chilled Omni Cryo unit filled with dry ice. 10 μl of RLT lysis buffer was added per 1 mg of heart tissue. 300 μl homogenate was used for RNA extraction. After elution, RNA concentration was measured using the NanoDrop ONE (Thermo Fisher Scientific) before dilution to 100 ng/μl in nuclease-free water (Thermo Fisher Scientific). Reverse transcription was performed using the High Capacity cDNA Reverse Transcription Kit (Applied Biosystems) in the Eppendorf Mastercycler X50s, using 1000 ng per reaction. cDNA solution was then diluted 1 in 4 with nuclease-free water. Control reactions without reverse transcriptase were performed alongside all experimental reactions.

Real-time quantitative PCR (RT-qPCR) was performed using TaqMan reagents on a LightCycler® 480 Instrument II (Roche) in duplex with minor groove binder (MGB) probes (Hein and Bodendorf, 2007). *Gapdh* was evaluated as a housekeeping control gene for normalisation using a 2′-chloro-7′phenyl-1,4-dichloro-6-carboxy-fluorescein (VIC)-coupled probe. Primer efficiency was calculated using a serial dilution of cDNA pooled from a group of WT animals and creating a dilution standard curve prior to experimental reactions as first described by Pfaffl (2001). All reactions were run in triplicates, and RT- and non-template controls were run on the same plate. Crossing point (Cp) values in qPCR were calculated by using the LightCycler 480 software with the Abs quant / 2nd derivative max function. Cp values for the gene of interest were normalised to duplexed *Gapdh* Cp values after adjusting for primer efficiency, as first described by Pfaffl (Pfaffl, 2001). Raw Cp values for the gene of interest and *Gapdh* were also visualised in all cases (e.g. Fig. 3J, Fig. S2E). TaqMan primer and probe mixes used for qPCR were purchased from Life Technologies and are listed in Table S3.

### Experimental and statistical analysis

Persons carrying out experiments and analyses were unaware of the genotype wherever possible, including all *in vivo* studies, tissue processing, RNA extraction, imaging and analysis, and unawareness was automated by an Excel template to prevent accidental memorisation of genotypes. Statistical analysis was performed using GraphPad Prism 9.2.0. Normal distribution was assumed for all data, as small numbers required by best practice in animal research preclude reliable testing for normality. Student's *t*-test was used for comparison of two groups and ANOVA for comparison of more than two groups. The Bonferroni correction was applied when multiple *t*-tests were performed. Šídák's multiple comparisons test was applied following ANOVA to data generated from the same animals at more than one timepoint. Tukey's multiple comparison test following ANOVA was used to compare values between multiple groups. Linear regression was used to compare two experimental groups for which the variable of interest depended on another variable that differed between groups (e.g. heart mass and body mass). *P*<0.05 was considered to be statistically significant. Data are presented as the mean±standard deviation (±s.d.).

## Supplementary Material

10.1242/dmm.050561_sup1 Supplementary information
