# Supplementary material for: Female Alms1-deficient mice develop echocardiographic features of adult but not infantile Alström syndrome cardiomyopathy
Source: Dis Model Mech. 2024 Jun 28;17(6):dmm050561. doi: 10.1242/dmm.050561 (PMC11225586; doi:10.1242/dmm.050561)
Supplement: Supplementary information [file dmm-17-050561-s1.pdf]

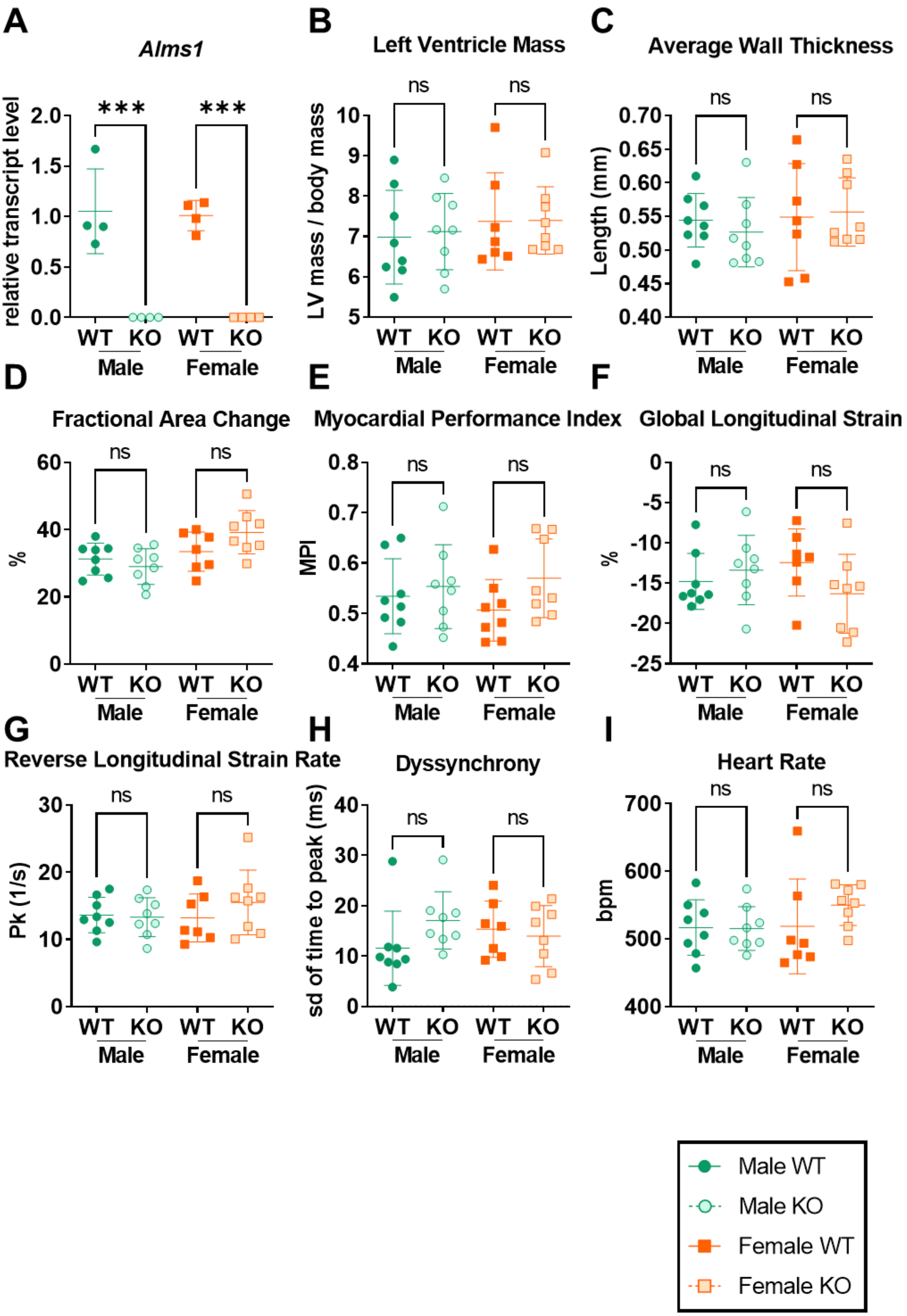

Disease Models & Mechanisms • Supplementary information

Fig. S1. Neither male nor female global *Alms1* knockout mice exhibit an echocardiographic phenotype at post-natal day 15. (**A**) qPCR confirmation of *Alms1* loss in heart tissue of global *Alms1* knockout (KO) mice with a Taqman probe for the 6-7 exon junction of *Alms1* **(B-I)** Echocardiography data. Left ventricle mass values (**B**) are normalised to total body mass. (**A**) Data presents ct values normalised to *Gapdh* run in duplex. Each data point represents an individual animal with bars representing mean ± sd. Comparison between groups in performed using two-way ANOVA with Tukey's multiple comparisons test. For (**A**) N = 4/group. For (**B-I**) N = 8, 8, 7 and 8 for WT males, KO males, WT females and KO females respectively.

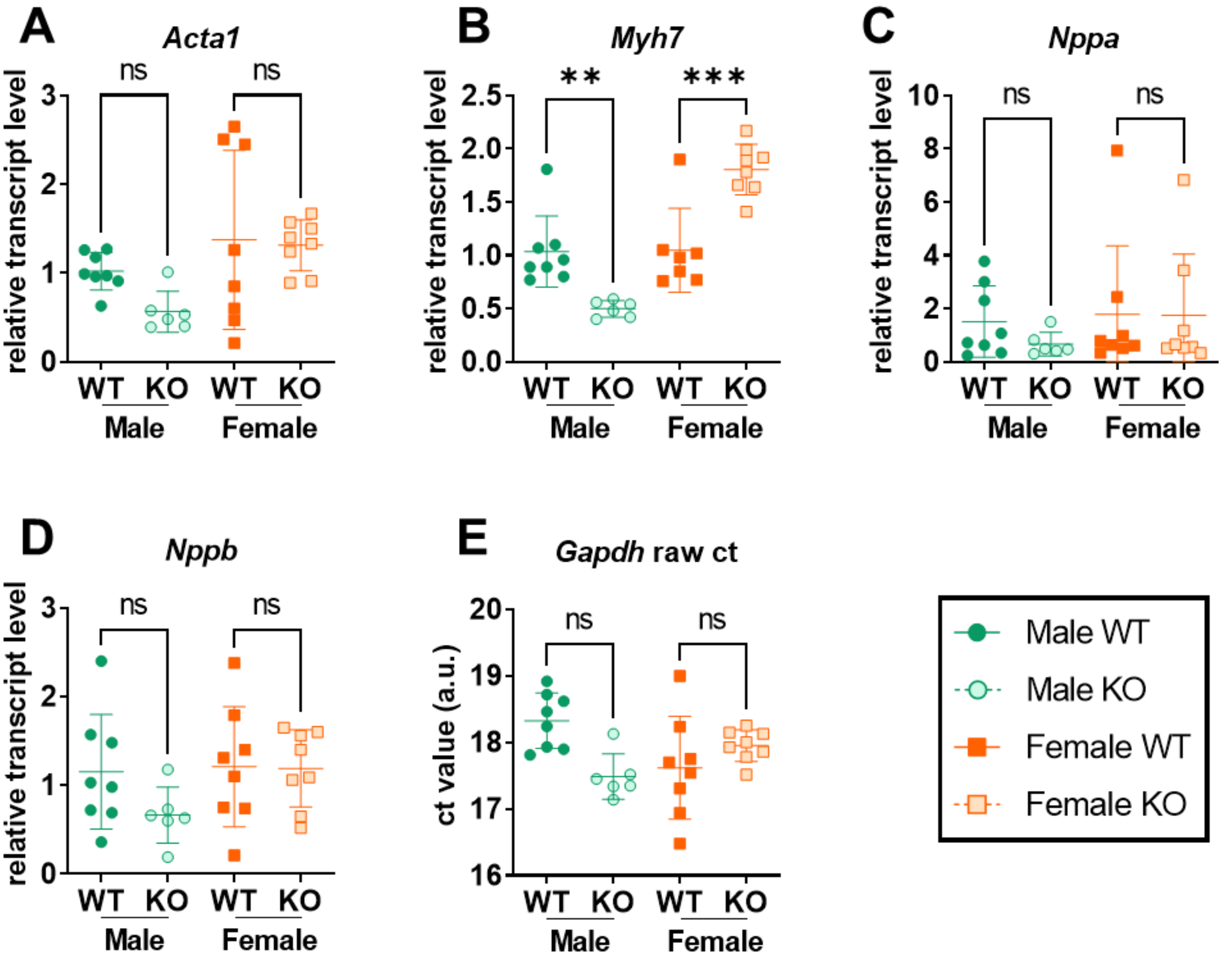

Fig. S2. Neither male nor female global *Alms1* knockout mice exhibit a cardiac transcriptional phenotype at post-natal day 15. (**A-D**) qPCR evaluation of heart tissue for typical markers of cardiomyopathy. Data presents ct values normalised to *Gapdh* run in duplex. (**E**) An example of raw *Gapdh* ct values from duplexed reactions. Each data point represents an individual animal with bars representing mean ± sd. Comparison between groups in performed using two-way ANOVA with Tukey's multiple comparisons test. N = 8, 8, 7 and 8 for WT males, KO males, WT females and KO females respectively.

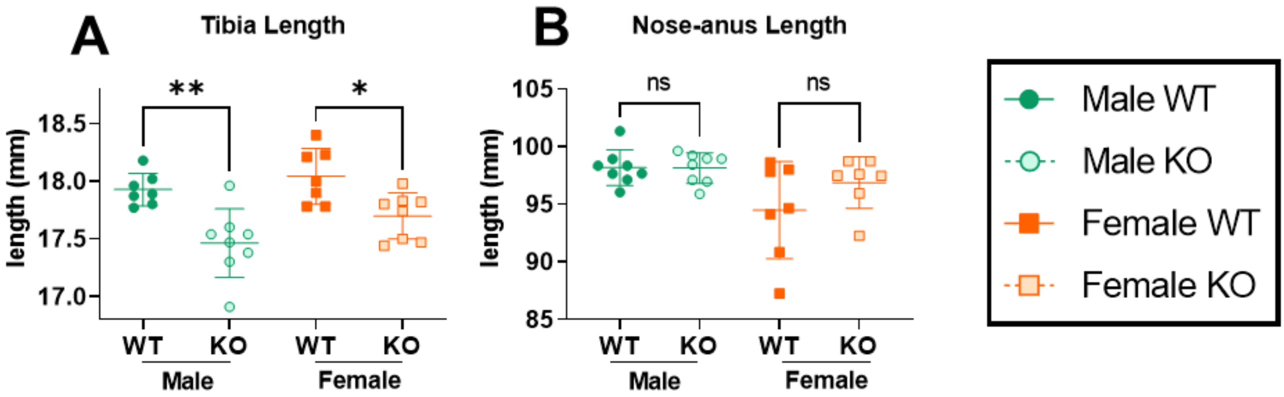

Fig. S3. Evaluation of parameters for normalisation of echocardiography area and mass values in adult global *Alms1* knockout mice. (**A**) Tibia length measured following cull at 24 weeks. (**B**) Nose-anus length measured in anaesthetised animals immediately following echo at 23 weeks. Each data point represents an individual animal with bars representing mean ± sd. Comparison between groups performed using two-way ANOVA with Tukey's multiple comparisons test. * denotes $p<0.05$ and ** denotes $p<0.01$. N = 8, 8, 7 and 8 for WT males, KO males, WT females and KO females respectively

Disease Models & Mechanisms • Supplementary information

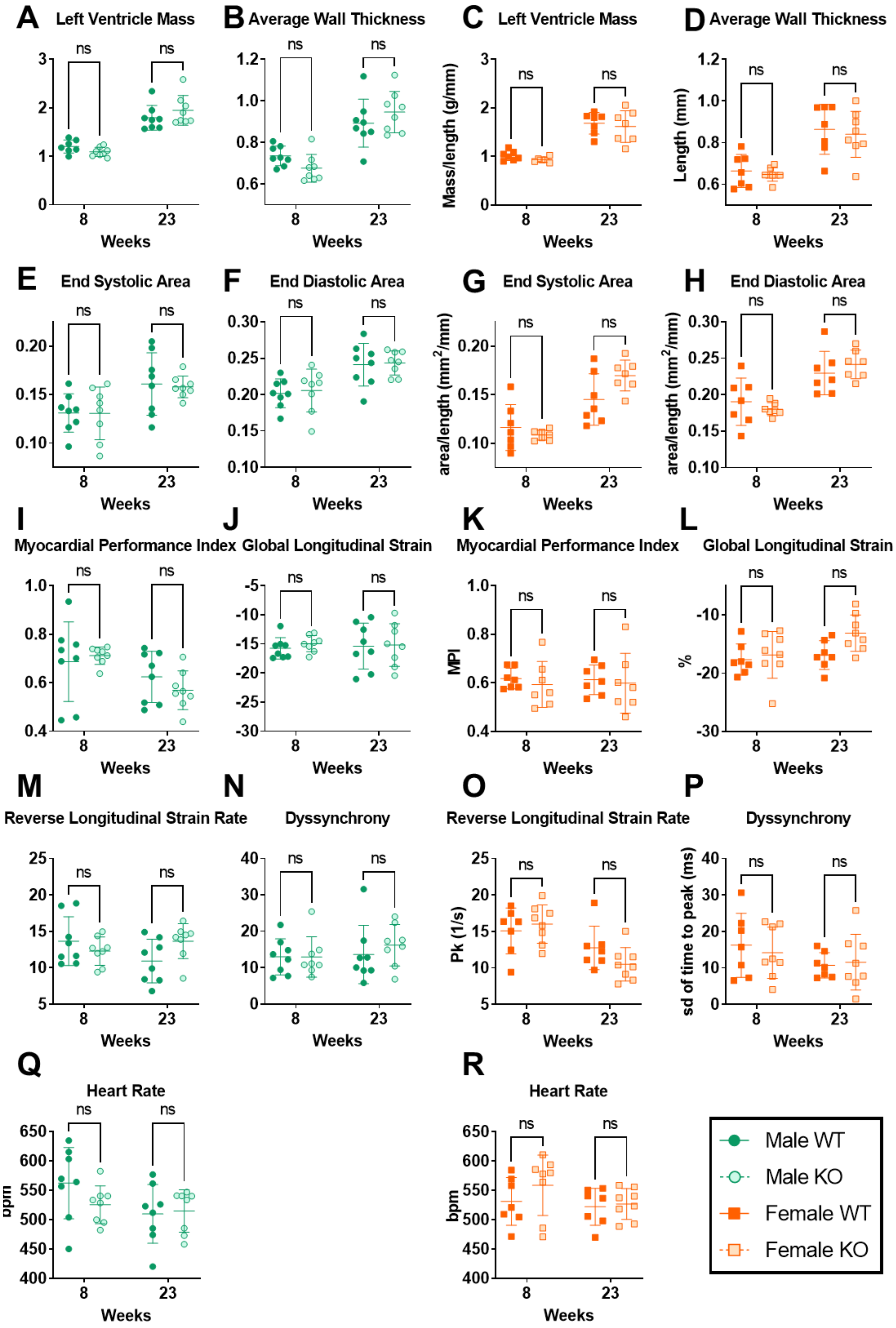
A
Left Ventricle Mass
B
Average Wall Thickness
C
Left Ventricle Mass
Mass/length (g/mm)
D
Average Wall Thickness
Length (mm)
E
End Systolic Area
F
End Diastolic Area
G
End Systolic Area
area/length (mm²/mm)
H
End Diastolic Area
area/length (mm²/mm)
I
Myocardial Performance Index
J
Global Longitudinal Strain
K
Myocardial Performance Index
MPI
L
Global Longitudinal Strain
%
M
Reverse Longitudinal Strain Rate
N
Dyssynchrony
O
Reverse Longitudinal Strain Rate
Pk (1/s)
P
Dyssynchrony
sd of time to peak (ms)
Q
Heart Rate
bpm
R
Heart Rate
bpm
ns
Weeks
8
23
Male WT
Male KO
Female WT
Female KO

Fig. S4. Systolic and diastolic dysfunction develops in female but not male global *Alms1* knockout mice with age. Echocardiography parameters measured at 8 and 23 weeks of age. Left ventricle mass and area values (**A,C,E-H**) are normalised to nose-anus length. Each data point represents an individual animal with bars representing mean ± sd. Comparison between groups performed using a two-way ANOVA with Šídák's multiple comparisons test. * denotes $p<0.05$ and ** denotes $p<0.01$. N = 8, 8, 7 and 8 for WT males, KO males, WT females and KO females respectively.

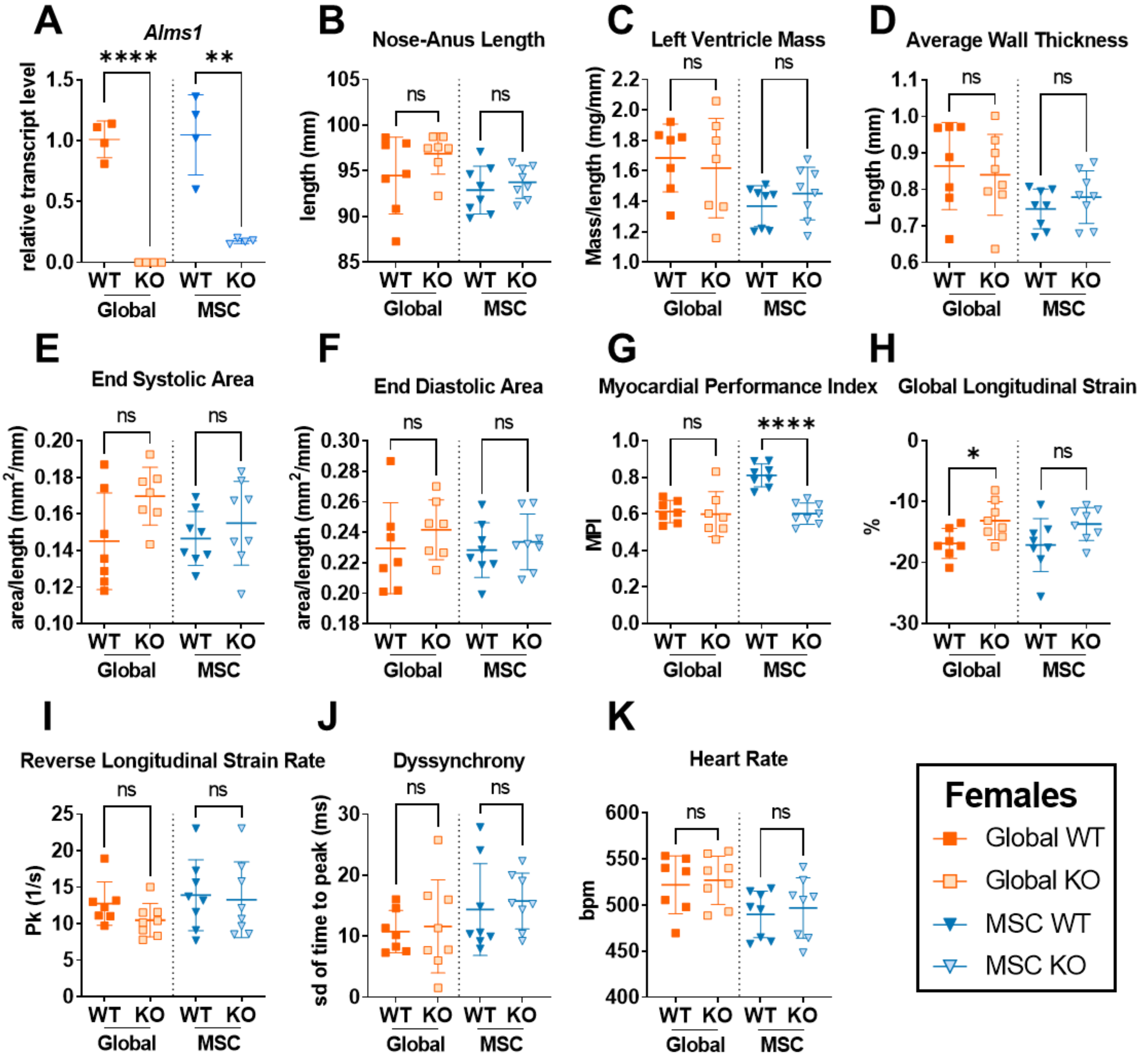

Fig. S5. Mesenchymal stem cell-specific *Alms1* knockout does not recapitulate the phenotype of global *Alms1* knockout. All global KO data repeated from Figure 2 and Fig. S4 for comparison to MSC-specific *Alms1* KO. (**A**) qPCR confirmation of partial *Alms1* loss in heart tissue of MSC-specific Alms1 KO mice with a Taqman probe for the 6-7 exon junction of *Alms1*. (**B**) Nose to anus length of females at 23 weeks of age. (**C-K**) Data obtained from analysis of echocardiography performed on female animals at 23 weeks of age. Mass and area values (**C,E,F**) normalised to nose-anus length. Each data point represents an individual animal with bars representing mean ± sd. Global WT/KO and MSC WT/KO experiments were

performed with identical design at different times; this is reflected in the dotted line separating the two cohorts. Comparison between WT and KO was performed using an unpaired two-tailed Student's t-test followed by a Bonferroni correction for multiple testing. * denotes $p<0.05$, ** denotes $p<0.01$, *** denotes $p<0.001$ and **** denotes $p<0.0001$. (**A**) N = 4/group (**B-K**) N = 7, 8, 8 and 8 for female global WT, global KO, MSC WT and MSC KO respectively.

Table S1. Reagents used for immunohistochemistry.

Item	Catalog No.	Company
Bond Epitope Retrieval ER1 Solution	AR9961	Leica Biosystems
Bond Epitope Retrieval ER2 Solution	AR9640	Leica Biosystems
Bond Wash Solution	AR9590	Leica Biosystems
Bond Polymer Refine Detection Kit	DS9800	Leica Biosystems
Normal Goat Serum	ab7481	Abcam
Mouse on Mouse Polymer IHC Kit	ab269452	Abcam
Rhodamine-conjugated Wheat Germ Agglutinin	RL-1022	Vector Laboratories
DAPI	D3571	Life Technologies
520 Green Opal reagent pack	FP1487001KT	Akoya
650 Blue opal reagent pack	FP1496001KT	Akoya

Table S2. Antibodies used for immunohistochemistry.

Antibody	Catalog No.	Species raised in	Company	Dilution
anti-cTnT (cardiac troponin)	MA512960	Mouse	Invitrogen, USA	1:500
Anti-Histone H3 (phospho S10)	ab5176	Rabbit	Abcam, UK	1:400
Goat F(ab) Anti-Rabbit IgG H&L (HRP)	ab7171	Goat	Abcam, UK	1:500

Disease Models & Mechanisms • Supplementary information

Table S3. TaqMan primer/probe mixes.

**Reagent**	**Gene target**	**Taqman Probe ID**	**Catalog No.**
Mouse GAPD (GAPDH) Endogenous Control (VIC™/MGB probe, primer limited)	*Gapdh*	Mm99999915_g1	4352339E
TaqMan™ Gene Expression Assay (FAM)	*Alms1* exon 6-7	Mm01189441_m1	4351372
TaqMan™ Gene Expression Assay (FAM)	*Acta1*	Mm00808218_g1	4331182
TaqMan™ Gene Expression Assay (FAM)	*Myh7*	Mm00600555_m1	4331182
TaqMan™ Gene Expression Assay (FAM)	*Nppa*	Mm01255748_g1	4331182
TaqMan™ Gene Expression Assay (FAM)	*Nppb*	Mm01255770_g1	4331182
TaqMan™ Gene Expression Assay (FAM)	*Col1a1*	Mm00801666_g1	4331182
TaqMan™ Gene Expression Assay (FAM)	*Lox*	Mm00495386_m1	4331182
TaqMan™ Gene Expression Assay (FAM)	*Cdkn1a*	Mm04205640_g1	4331182
TaqMan™ Gene Expression Assay (FAM)	*Cdkn2a*	Mm00494449_m1	4331182
TaqMan™ Gene Expression Assay (FAM)	*Lmnb1*	Mm00521949_m1	4331182

Disease Models & Mechanisms • Supplementary information
